# Development and evaluation of formulations of microbial biotransformed extract of tobacco leaves for hair growth potential

**DOI:** 10.4103/0974-8490.72328

**Published:** 2010

**Authors:** Ashlesh V. Murkute, Mahesh S. Sahu, Prashant Y. Mali, Vinod D. Rangari

**Affiliations:** *Department of Pharmacognosy, J. L. Chaturvedi College of Pharmacy, Nagpur-440016 (M.S), India*; 1 *Department of Pharmacognosy, Radharaman Institute of Pharmaceutical Sciences, Ratibad, Bhopal - 462 044 (M.P.), India*; 2 *Department of Pharmacology, Radharaman College of Pharmacy, Ratibad, Bhopal-462044 (M.P.), India*

**Keywords:** Alopecia, hair growth, tobacco leaves

## Abstract

**Background:**

Extensive researches are going on to explore the effective and safe drug for their hair growth. Tobacco leaves are traditionally known to potentiate hair growth promotion. Therefore, the aim of present study was to formulate and evaluate the microbial biotransformed extract of tobacco leaves for hair growth potential in male albino wister rats.

**Materials and Methods:**

The extract of was prepared by microbial biotransformation of tobacco leaves in cow urine for 28 days. The herbal formulations (lotion) were formulated by general method using o/w type base in various rations or concentrations such as 10%, 20% and 30% of extract. These lotions were applied on shaved skin area of rats for 30 days once in a day and hair length, serum total protein, and total testosterone were measured.

**Results:**

Our formulations show increase in hair growth and serum total protein at concentration dependent manner with effect to standard and control groups. Serum total testosterone decreases according to a concentration dependent manner.

**Conclusion:**

Further, series of investigations are, however, necessary to remain exploration, which includes their structural elucidation, characterization, clinical safety, reliability and molecular mechanism involved in this pharmacological activity.

## INTRODUCTION

Hairs are considered to be a major component of an individual’s general appearance. Hair loss (alopecia) creates the psychological impact and results in a measurably change in self esteem. Angiogenesis (through endogenous substances), androgen antagonism, potassium channel opening, and 5-alpha reductase inhibition are the major non-surgical therapeutic strategies of hair growth promotion.[1] Hairs are important sociologically and make the vital part of a human personality. Hair disorder, especially when severe, often profoundly affects the lives of those afflicted. Alopecia is a dermatological disorder that has been recognized for more than 2000 years. It is a common and distressing problem in cosmetics as well as primary health practice. It is common throughout the world and has been estimated to affect about 2% of the world population.[2–4] Alopecia also results in reduced social interactions in school age children and teenagers.[5–7] Alopecia affects approximately 50% of men over 40 years of age and also affect just as many as women. The majority of men and women (90%) or more want to reverse half hair loss. Alopecia is a synonym of baldness involve absence or loss of hair especially of the head. Androgens are well known to cause regression and balding on the scalp in genetically disposed individuals. Alopecia has also been observed as a major side effect of anticancer drugs, immunosuppressant, and many other drug treatments.[8] Currently, minoxidil (useful in both male and female pattern baldness) and finasteride (useful in male pattern baldness) are two U.S. FDA approved synthetic drugs finding concomitant use for treatment of androgenic alopecia, but their side effects have reduced their usage.[9,10] Hormone therapy use alpha-reductase inhibitors in the treatment of alopecia.[11] Though the side effects associated with this drugs have limited its pharmacological benefits. Hence, the drug of plant origin is necessary to replace the synthetic one. India is repository of medicinal plants.[12,13] Besides healthcare, the herbs are used in beatification of body and for preparation various cosmetics.[14] In traditional system of medicine, many plants and herbal formulation are reported for hair growth promotion.[15–19] *Nicotiana tabaccum* (Leguminosae) commonly known as tobacco or in Hindi Tamakhu. Tobacco leaves posses narcotic, sedative, emetic, carminative, anthelmintic etc properties. The leaves also useful in the treatment of bronchitis, asthma, cancer of teeth, skin diseases, scorpion sting, headache, chronic giddiness, and ranting.[20–24] The principal constituent of tobacco leaves is the alkaloid nicotine. They also contain a crystalline substance nicotianin and small quantities of alkaloids other than nicotine viz. nicotinine, nicoteine and nicoteline together with traces of a volatile oil, etc.[25]

Hence, the present study is an effort to formulate and evaluate the microbial biotransformed extract of tobacco leaves for hair growth potential selected on the basis of traditional use and evidence of microorganism responsible for biotransformation of leaves in cow urine.

## MATERIALS AND METHODS

### Plant material and extraction

Fresh leaves of tobacco were collected in the month of August locally from the Nagpur. The plant and leaves were authenticated by a pharmacognocist Dr. Vinod D. Rangari, Department of Pharmacognosy, J. L. Chaturvedi College of Pharmacy, Nagpur-440016 (MS), India. The leaves were dried under shade and macerated with cow urine for 28 days with occasional stirring. After filtration with muslin cloth, solvent was removed by distillation under vacuum.[26] The crude residue mass of extract were concentrated, stored and preserved (2-8° C). It is considered as a microbial biotransformed extract.

### Chemicals and reagents

Minoxidil [Mintop, 2% lotion] (Dr. Reddy’s Lab, Hyderabad) and all other diagnostic kits and solvents used for experimental works were of AR grade.

### Animals

Male albino wister rats (120-150 g) were used. The animals were fed with standard pellet diet and water *ad libitum* and maintained under standard environmental conditions (22°C ± 5 °C with 12 h of light-dark cycle). All experimental protocols were approved by Institutional Animal Ethical Committee Clearance (JLCCP/IAEC, 2007/2/CPCSEA), J. L. Chaturvedi College of Pharmacy, Nagpur-440016(M.S), India.

### Microbial study

The isolation of microorganisms from the extract was done by using Streak Plate Technique.[27] These isolated microorganism culture were subjected to Disha Biotech Lab, Nagpur, for their identification having wide sample reference no. E07A187.02A (1141 and 1142) at dated 18 Oct, 2007.

### Preparation of formulations

Herbal lotions were prepared by general method using o/w type base. The formula of base contains lanoline (5% w/w), cetyl alcohol (3% w/w), bees wax (2% w/w), propylene glycol (1.5% w/w), sesame oil (1.5% w/w), stearic acid (2% w/w), preservative (q.s.), perfume (q.s.) is considered as phase-A, commonly for all three lotion preparations (10%, 20% and 30%). Phase-B was made by various concentrations of extract such as 10% w/w, 20% w/w, and 30% w/w by making volume upto 100 ml with the help of distilled water. After preparation of both phases, phase-A was added slowly in phase-B by continuous triturating till uniform consistency of lotion was attained.[28]

### Hair growth activity

The rats were divided into five groups of six rats each. A 4 cm ^2^ area of dorsal portion of all rats were shaved and wiped with surgical spirit. Hair remover was also applied over the shaved area to assure the removal of trace of hairs. The animals in Group I, considered as control, Group II treated as standard. Applied 2% Minoxidil lotion over the shaved area once a day. Group III, IV and V were considered as treatment groups, Application of 10%, 20% and 30% lotion formulations, respectively. The treatment was continued for 30 days.[29] On day 15 and 30 of the treatment, hairs were plucked randomly from the shaved area of selected rats and length of 24 hairs was measured by vernier caliper (Mitutoyo Digimatic).[30] The average length was determined. On day 30, blood was collected from retro-orbital plexus, and serum total protein (Modified Biuret Method) and total testosterone (Samples was tested from Dr. LalPath Labs Pvt. Ltd., Nagpur). Physical parameter such as hair length has been considered as vital accomplishment for hair growth and biochemical parameters accomplish clinical manifestations of alopecia as well as development of hairs.

### Statistical analysis

Data were expressed as the mean standard deviation of the means (S.D.) and statistical analysis was carried out by employing student *t*-test. *p*<0.05 was considered as statistical significant.

## RESULTS AND DISCUSSION

### Microbial study

After identification, the *Pseudomonas aeruginosa* (E07A187.02A (1141)) and *Enterococus avium* (E07A187.02A (1142) microorganisms were found in the extract culture, which are responsible for the biotransformation of tobacco leaves in cow urine.

### Hair growth activity

Upon pondering the causes of hair loss, it becomes clear to search and plan the strategies for hair growth. Natural products are very popular and well accepted in the cosmetic and hair care industries and about 1000 plant extracts have been examined for hair care usage. There are many products available in the market, which are prepared by combination of one or more herbal drugs and find acceptability as hair tonic, hair growth promoter, hair conditioner, hair cleansing agent, antidandruff agents, and for the treatment of alopecia and lice infections.[12,31] Therefore, the results of our study revealed that, there is significant increase in hair length and total protein of standard group animals at 30 day of treatment as compared to control group as shown [Tables 1 and 2]. However, hair length and total protein in treatment groups incresesed significantly at concentration dependent manner in all three formulations with effect to standard. It is clear that our extract formulations act as a vital accomplishment for hair growth. An analysis of hair shows, it is composed of iron, oxygen, hydrogen, nitrogen, and sulfur. The blood must be supplied with these minerals so that nourishment will be carried to the scalp. Angiogenesis, the formation of new blood vessels from preexisting vascular network, is a driving force of hair growth. Therefore, modulation of angiogenesis is considered as therapeutic strategies of great importance for hair growth.[32] Niacin (Vitamin B_3_), Vitamin B complex, ascorbic acid (Vitamin C), Tocoferol (Vitamin E), Zinc, essential fatty acids (primose and salmon oil, etc.), amino acids (L-cysteine & L-methionine) are some of the various nutrients and minerals, which play important role in preventing hair loss. Although nutrition does play a role in hair loss and in the overall health of your hair, only extreme nutritional deficiencies will cause hair loss.[1] The total testosterone level in treatment groups decrease significantly at concentration dependent manner in all three formulations with respect to standard group as shown [Table 2]. The total testosterones in scalp are produced by androgen hormones and is converted into dihydrotestosterone by the enzyme 5-alpha reductase. Increased levels of dihydrotestosterone in the scalp reduce blood supply, which are critical to growth of new hair.[33] The mechanism of our standard drug minoxidil promote hair growth is not fully understood. Minoxidil is a potassium channel opener, causing hyperpolarization of cell membranes. Minoxidil is less effective when there is a large area of hair loss. In addition, its effectiveness has largely been demonstrated in younger men (18-41 years of age). Minoxidil use is indicated for central (vertex) balding only.[34] The hair growth potential of our microbial biotransformed extract of tobacco leaves may be due to alkaloids such as nicotine, nicotianin, and other constituent’s viz. nicotinine, nicoteine and nicoteline which are responsible for selectively inhibiting 5-alpha reductase activity. Further, series of study require on this microbial biotransformed extract of tobacco leaves in cow urine for the betterment of mankind in treating various ailments.

**Table 1:** Effect of various formulations of biotransformed extract of tobacco leaves on hair length in rats

Treatment groups	Hair length (Mm)	
	15 days	30 days
Control	3.33 ± 0.082	6.07 ± 0.122
Standard	4.47 ± 0.096	9.17 ± 0.146*
10% lotion	3.73 ± 0.016	7.73 ± 0.321*
20% lotion	3.86 ± 0.118*	8.98 ± 0.435**
30% lotion	4.26 ± 0.296*	9.26 ± 0.778**

Data were expressed as the mean standard deviation of the means (S.D.) and statistical analysis was carried out by employing student *t*-test. *p*<0.05 was considered as statistical significant, *n* = 6 in each group.

**Table 2:** Effect of various formulations of biotransformed extract of tobacco leaves on serum total protein and total testosterone in rats

Treatment Groups	Total protein (mg/dl)	Total Testosterone (ng/ml)
Control	5.56 ± 0.219	4.14 ± 0.057
Standard	7.02 ± 0.521**	3.54 ± 0.057**
10% lotion	6.17 ± 0.601*	4.02 ± 0.080*
20% lotion	6.54 ± 0.582*	3.71 ± 0.078*
30% lotion	6.98 ± 0.592**	3.59 ± 0.033**

Data were expressed as the mean standard deviation of the means (S.D.) and statistical analysis was carried out by employing student *t*-test. *p*<0.05 was considered as statistical significant, *n* = 6 in each group.

## CONCLUSION

Hence, it can conclude that, the microbial biotransformed extract of tobacco leaves in cow urine increases hair growth at concentration-dependent manner in all three formulations with effect to standard and control. These formulations were also studied or tested on chemotherapy-induced alopecia in human volunteers. Therefore, extract formulations may require their structural elucidation, characterization, clinical safety, reliability, and molecular mechanism remain exploration, which would give a positive lead in treating various diseases in feature.
